# Supplementary material for: The genome sequence of the highfin lizardfish, Bathysaurus mollis Günther, 1878 (Aulopiformes: Bathysauridae)
Source: Wellcome Open Res. 2025 Oct 24;10:605. [Version 1] doi: 10.12688/wellcomeopenres.25077.1 (PMC12640494; doi:10.12688/wellcomeopenres.25077.1)
Supplement: Peer review history

# Reviewer report: Reviewer response for version 1

**DOI:** 10.21956/wellcomeopenres.27644.r138505

**Published:** 2025-11-27

**Authors:** Salvatore D'Aniello, Anna Albanese

**Affiliations:** 1 Stazione Zoologica Anton Dohrn, Napoli, Italy; 2 Universita degli Studi di Ferrara Dipartimento di Scienze della Vita e Biotecnologie, Ferrara, Emilia-Romagna, Italy

The present manuscript regards the genome sequencing of highfin lizardfish *Bathysaurus mollis,* whitin the Darwin Tree of Life iniciative.

 We believe that the quality of the experimental protocols and computational analyses are highly standardized and in general of high quality.

 The present project will provide material for comparative genomics studies, and therefore we support its publication.

 We would ask to the authors to revise the spectrophotometric values of nucleic acid extraction, since they declare very high values: the 260/280 ratio was 2.98, and the 260/230 ratio was 6.41.

Are sufficient details of methods and materials provided to allow replication by others?

Yes

Is the rationale for creating the dataset(s) clearly described?

Yes

Are the datasets clearly presented in a useable and accessible format?

Yes

Are the protocols appropriate and is the work technically sound?

Yes

Reviewer Expertise:

Evolutionary and Developmental Biology (EvoDevo)

We confirm that we have read this submission and believe that we have an appropriate level of expertise to confirm that it is of an acceptable scientific standard.

# Reviewer report: Reviewer response for version 1

**DOI:** 10.21956/wellcomeopenres.27644.r138504

**Published:** 2025-11-25

**Authors:** Anthony K. Redmond

**Affiliations:** 1 University College Dublin, Dublin, Ireland

The study encompassing production of a genome assembly and raw RNA-seq data for the highfin lizardfish. The study appears to be performed to a high standard and data appropriately shared openly. The genome assembly appears to be of good accuracy and completeness. I am overall please with the study and have no major comments.

Are sufficient details of methods and materials provided to allow replication by others?

Yes

Is the rationale for creating the dataset(s) clearly described?

Yes

Are the datasets clearly presented in a useable and accessible format?

Yes

Are the protocols appropriate and is the work technically sound?

Yes

Reviewer Expertise:

genome evolution

I confirm that I have read this submission and believe that I have an appropriate level of expertise to confirm that it is of an acceptable scientific standard.

# Reviewer report: Reviewer response for version 1

**DOI:** 10.21956/wellcomeopenres.27644.r138508

**Published:** 2025-11-22

**Authors:** Yichen Dai

**Affiliations:** 1 Fudan University, Shanghai, Shanghai, China

The Darwin Tree of Life project is extremely important for advancing our understanding of evolution from a molecular perspective. The sampling and assembly of different eukaryotic species is a huge effort, not to also mention that the quality of these assembled genomes is extremely high. Specifically for this paper, the authors present a genome assembly for *Bathysaurus mollis*, which is an interesting resource for those investigating evolution of the fish lineage. I have no issues with the manuscript, and the methods and genome assembly described in the manuscript are detailed. In addition, I have verified that the genome data submitted are openly accessible. My only suggestion for the authors is that it would be very helpful to the readers if they could include photos or a figure when they describe the difference between *Bathysaurus mollis* and *B*. *ferox*.

Are sufficient details of methods and materials provided to allow replication by others?

Yes

Is the rationale for creating the dataset(s) clearly described?

Yes

Are the datasets clearly presented in a useable and accessible format?

Yes

Are the protocols appropriate and is the work technically sound?

Yes

Reviewer Expertise:

Genome evolution, molecular evolution, evo-devo

I confirm that I have read this submission and believe that I have an appropriate level of expertise to confirm that it is of an acceptable scientific standard.

# Reviewer report: Reviewer response for version 1

**DOI:** 10.21956/wellcomeopenres.27644.r138509

**Published:** 2025-11-12

**Authors:** David Ray

**Affiliations:** 1 Texas Tech University, Lubbock, Texas, USA

This is a standard data note for the Darwin Tree of Life project. All of the typical information is present and clearly presented. 

 I note only one small problem. Near the end of the Nucleic acid extraction paragraph, a number is missing before 'kb'. This should indicate the mean fragment size.

Are sufficient details of methods and materials provided to allow replication by others?

Yes

Is the rationale for creating the dataset(s) clearly described?

Yes

Are the datasets clearly presented in a useable and accessible format?

Yes

Are the protocols appropriate and is the work technically sound?

Yes

Reviewer Expertise:

Vertebrate genonimcs and evolution

I confirm that I have read this submission and believe that I have an appropriate level of expertise to confirm that it is of an acceptable scientific standard.
